# Computational investigation of the global prevalence of multidrug resistant *Mycobacterium leprae*: A systematic review and meta-analysis

**DOI:** 10.1016/j.jctube.2024.100495

**Published:** 2024-11-07

**Authors:** Hamidreza Zivarifar, Forough Ahrari, Mohsen Karbalaei

**Affiliations:** a Department of Internal Medicine, and Virology, School of Medicine, Zahedan University of Medical Sciences, Zahedan, Iran; b Clinical Immunology Research Center, Ali-Ebne Abitaleb Hospital, Zahedan University of Medical Sciences, Zahedan, Iran; c Department of Family and Community Medicine, School of Medicine, Birjand University of Medical Sciences, Birjand, Iran; d Department of Microbiology and Virology, School of Medicine, Jiroft University of Medical Sciences, Jiroft, Iran; e Bio Environmental Health Hazards Research Center, Jiroft University of Medical Sciences, Jiroft, Iran

**Keywords:** Drug resistance, Meta-analysis, *Mycobacterium leprae*, Prevalence

## Abstract

**Background:**

Leprosy is a chronic infectious disease caused by *Mycobacterium leprae* (*M. leprae*) However, the emergence of drug-resistant strains of this bacterium, especially multidrug-resistant (MDR) strains, is a serious concern. This study aimed to evaluate the global prevalence of MDR *M. leprae* and its implications.

**Methods:**

Using PRISMA guidelines, we systematically reviewed ISI Web of Science, MEDLINE, and EMBASE up to August 2023 to assess the prevalence of MDR *M. leprae*. We included human clinical trials on MDR *M. leprae*, as well as excluded reviews, animal studies, and unavailable full texts. Data was analyzed using Comprehensive Meta-Analysis software, and publication bias was addressed using Egger’s, Begg’s tests, and the trim-fill method.

**Results:**

Overall, 861 articles were initially identified, of which 28 met the methodological criteria for inclusion in the quantitative synthesis. Statistically, the combined prevalence of drug resistant *M. leprae* was approximated at 11.7 % (95 % CI: 7.7–17.3; *I^2^*: 90.79; *p* value = 0.01). Specific drug resistance rates included 7.4 % to dapsone and 5.1 % to rifampin, among others. The global rate for MDR *M. leprae* was measured at 2.2 % (95 % CI: 1.2–3.9; *I^2^*: 82.68; *p* value = 0.01). Factors such as bacterial density and the lepromatous phase were associated with elevated DR *M. leprae* risk (OR: 2.69; 95 % CI: 1.35–2.48). A systematic assessment of publication bias indicated a minimal impact on the general results.

**Conclusions:**

The increasing prevalence of MDR *M. leprae* globally requires urgent and strategic interventions to prevent further spread, which in turn is effective in treating leprosy patients.

## 1 Background

*Mycobacterium leprae* (*M. leprae*) is the causative agent of leprosy, a chronic infectious disease that primarily affects the skin and peripheral nerves. It is a neglected tropical disease that has been documented for many centuries preceding the biblical era [1]. This infectious disease is a significant public health concern, with more than 200,000 new cases reported every year in over 120 countries [2]. If left untreated, the disease can cause permanent damage to the skin, nerves, limbs, nose, and eyes, leading to disabilities in a small proportion of patients [3]. Although major countries endemic for leprosy in 1985 have reached the elimination target and several countries have achieved the “elimination” level, but new case detection continues unabated, leprosy remains a major public-health problem in nine countries in Africa, Asia, and Latin America [4].

The World Health Organization (WHO) has developed a global strategy of multidrug therapy (MDT) for treating leprosy; 3-drug regimen that includes rifampicin, dapsone, and clofazimine. Paucibacillary leprosy (PB), characterized by a low bacterial load and a restricted number of skin lesions, requires 6 months of treatment, whereas multibacillary leprosy (ML) requires 12 months [5]. Although rifampicin is a key component of the recommended regimen for leprosy treatment, resistance to this drug can lead to treatment failure and disease progression [6]. Patients have shown different levels of resistance in different regions, with the highest incidence observed in the Western Pacific. In a meta-analysis conducted by the WHO during 2009–2015, resistance to rifampicin was shown without a clear increasing trend. Unfortunately, rifampicin-resistant *M. leprae* was twice more in patients with leprosy recurrence [6], [7].

Furthermore, alternative treatment options such as clarithromycin, minocycline, and quinolones may be considered in cases of rifampicin resistance [8]. But prolonged, interrupted and inadequate use of antibiotic monotherapy such as dapsone can lead to the relatively widespread presence and spread of drug resistance in leprosy [9]. Also, genetic mutations have been observed in cases of drug-resistant *M. leprae* (DR *M. leprae*)such as mutations in the *folP*, *rpoB*, and *gyr* genes, which are associated with resistance to dapsone, rifampicin, and ofloxacin, respectively [10], [11]. Therefore, understanding the global prevalence of multidrug-resistant (MDR) *M. leprae* is essential to guide treatment strategies and surveillance efforts. Our systematic review and meta-analysis study will provide a comprehensive overview of the current state of drug resistance in leprosy and identify factors associated with increased resistance. By identifying the regions and populations at higher risk of MDR *M. leprae*, targeted interventions can be developed to prevent the spread of drug resistance and ensure effective treatment for all leprosy patients.

## 2 Methods

### 2.1 Search strategy

This study was designed according to the guidelines of the Preferred Reporting Items for Systematic Reviews and Meta-Analyses (PRISMA) checklist [12]. In this study, a meta-analysis was conducted to gather all potentially relevant publications. This was achieved using a comprehensive search of computer-assisted databases such as ISI Web of Science, MEDLINE, and EMBASE. Articles were collected regardless of language or publication date restrictions. Two independent authors were engaged in this process, wherein the relevant studies were searched using various keywords based on MeSH. These keywords included “*Mycobacterium leprae*”, “*M. leprae*”, “Leprosy”, “Antimicrobial resistance”, “Drug resistance”, “Multidrug resistant”, “MDR *M. leprae*”, “Mutation”, “*folp1*″, ”*rpoB*“, ”*gyrA*“, and ”*gyrB*“. We attempted to limit the search to human clinical trials. The literature review procedure was conducted till August 2023. Furthermore, a manual search of bibliographic lists was conducted to discover any further research that may have been missed.

### 2.2 Selection criteria

The procedure was conducted with the involvement of two separate authors. The inclusion criteria encompassed studies that investigated the presence of MDR *M. leprae*. Studies with the following criteria were included in the meta-analysis: 1) cross-sectional studies that evaluated the prevalence of MDR *M. leprae*; 2) clinical trials involving human subjects; 3) investigations that examined drug resistance in *M. leprae* utilizing precise methodologies; 4) studies that provided comprehensive data, including the number of both drug-resistant and drug-susceptible *M. leprae* strains. Whereas, review articles, letters, case reports, duplicate cases, studies conducted on animals, studies with insufficient methods and findings, studies on non-human subjects, as well as studies without available full text.

### 2.3 Data extraction and quality assessment

We used the Joanna Briggs Institute (JBI) checklist to assess the quality of studies that emphasize critical aspects, e.g., population size, research objectives, sample collection, and statistical methods [13]. One score was assigned to each parameter; in this relation, each study was included if at least six scores were achieved. This checklist evaluates many factors such as population size, study objectives, sample collection method, and statistical analysis. All researches that obtained a minimum of six scores were considered eligible for inclusion. The provided information included: 1) first author; 2) year of publication; 3) geographical location of the conducted studies. The following information was retrieved from qualifying studies: 1) the number of positive samples for *M. leprae* 2) the percentage of resistance to dapsone, rifampin, clofazimine, and ofloxacin, including drug-resistant strains; 3) the prevalence of MDR *M. leprae*. In this context, the geographical areas of study were determined based on the classification of regions established by the WHO. This section involved the collaboration of two different authors. However, any issues that arose between the two investigators were successfully resolved through conversation.

### 2.4 Statistical analysis

We pooled the data using Comprehensive Meta-Analysis (CMA) software version 2.2 (Biostat, Englewood, NJ, USA). Both event rate and 95 % confidence intervals (CI) were used to measure antibiotic resistance in *M. leprae* as well as the prevalence of MDR *M. leprae*. Additionally, the risk factor associated with drug resistance of *M. leprae* was assessed by calculating the odds ratio (OR) with 95 % CI. Subsequently, a subgroup analysis was conducted according to the therapeutic states, duration of the course, and WHO’s area classification. In addition, a sensitivity analysis was performed to examine potential sources of bias in the included studies. The assessment of heterogeneity was conducted using *I^2^* statistics and the Cochrane *Q p* value. Using the Der-Simonian and Laird technique, a Random-effects model was employed when significant heterogeneity was observed (*I^2^* > 50 % and Cochrane *Q p* value > 0.05). The assessment of publication bias was conducted using Egger’s and Begg’s *p* value tests. In addition, the presence of publication bias was explored by examining the asymmetry of the funnel plot. The trim-fill method was used as a means of addressing bias in states where there was a significant publication bias.

## 3 Results

### 3.1 Literature search outcomes

**Fig. 1:**
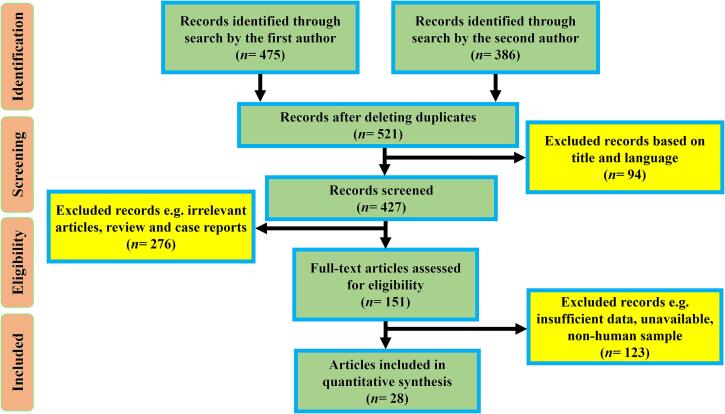
Flow chart of literature screening.

Through a comprehensive database search, we retrieved 861 potential articles. After carefully reviewing the titles, abstracts, and full texts, 833 articles were excluded from the study. Finally, 28 articles that demonstrated high methodological quality met the inclusion criteria for the present quantitative synthesis (Table 1) [14], [15], [16], [17], [18], [19], [20], [21], [22], [23], [24], [25], [26], [27], [28], [29], [30], [31], [32], [33], [34], [35], [36], [37], [38], [39], [40], [41]. The selection process is delineated in accordance with the PRISMA guidelines (Fig. 1).

**Table 1:** Characteristics of included studies.

First author	Year	Country	*M. leprae* cases	Prevalence of resistance (%)						Diagnostic method	JBI score	Ref
				DR	MDR	Dapsone	Rifampin	Clofazimine	Ofloxacin			
Maeda	2001	Asia	88	14.7	3.4	4.54	3.4	ND	1.13	Mouse footpad assay	8	[14]
Sekar	2002	India	214	ND	0.46	16.3	0	0.46	ND	Mouse footpad assay	6	[15]
Ebenezer	2002	India	369	6.23	0.27	4.88	1.36	2.44	ND	Mouse footpad assay	7	[16]
Kim	2003	Korea	7	100	14.2	57.14	28.57	ND	14.2	Sequencing	7	[17]
You	2005	Korea	104	ND	5	19.2	2.88	ND	0.96	Sequencing	6	[18]
Matsuoka	2007	Asia	305	ND	0	4.58	11.54	ND	0.00	Sequencing	6	[19]
Ozaki	2008	Japan	36	ND	6	52.7	39.3	ND	19.35	Sequencing	8	[20]
Matsuoka	2010	Japan	38	5.26	2.63	0.0	5.26	ND	2.63	Sequencing	7	[21]
Kai	2011	Vietnam	290	0	0	10.1	4.2	ND	0.0	Sequencing	7	[22]
Singh	2011	Latin America	233	1.28	0	0.86	0.43	ND	0.0	Sequencing	6	[23]
Rocha	2012	Brazil	57	7.01	3.50	5.26	7.01	ND	3.50	Sequencing	8	[24]
Williams	2013	USA	39	7.69	0	5.13	2.56	ND	0.0	Sequencing	8	[25]
Guerrero	2014	Colombia	941	4.14	0.42	0.21	2.65	ND	0.63	Sequencing	7	[26]
Lavania	2014	India	111	ND	1.80	8.11	3.60	ND	9.01	Sequencing	6	[27]
Contreras	2014	Brazil	197	ND	1.31	ND	5.26	ND	ND	Sequencing	8	[28]
Liu	2015	China	85	25.9	3.70	1.5	8.8	ND	25.9	Sequencing	7	[29]
Lavania	2015	India	215	ND	0	0.47	3.26	ND	0.0	Sequencing	7	[30]
Alzate	2016	Colombia	134	7.46	0.74	2.98	2.98	ND	0.74	Sequencing	6	[31]
Avanzi	2016	Africa	24	12.5	0	12.5	4.17	ND	0.0	Sequencing	7	[32]
Lavania	2017	India	250	28.4	6.0	7.2	10.4	ND	4.0	Sequencing	7	[33]
Chauffour	2018	France	160	11.25	0	8.1	1.8	ND	1.25	Sequencing	7	[34]
Benjak	2018	Multi-center	154	24.04	16.23	15.58	14.28	ND	6.49	Sequencing	6	[35]
Singh	2018	India	78	10.25	0.0	0.0	0.0	ND	32.0	Sequencing	8	[36]
Chen	2019	China	76	19.0	1.31	25.0	0	ND	1.31	Sequencing	6	[37]
Chokkakula	2019	China	290	NR	2.0	2.76	0.34	ND	2.76	Sequencing	6	[38]
Rosa	2020	Brazil	37	43.24	32.43	40.54	35.13	ND	ND	Sequencing	7	[39]
Mahajan	2020	India	77	7.79	2.59	2.59	1.29	ND	6.49	Sequencing	7	[40]
Narang	2021	India	61	16.39	3.27	8.19	9.83	ND	2.70	qPCR-HRM	6	[41]

### 3.2 Characteristics of the selected studies

All 28 included studies examined the prevalence of MDR *M. leprae* from 2001 to 2021. These studies were regionally distributed as follows: 8 studies from the WHO Western Pacific Region (WPR), 10 studies from the WHO South-East Asia Region (SEAR), 7 studies from the WHO Region of the Americas (AMR), 1 study from the WHO European Region (EUR), 1 study from the WHO African Region (AFR), and 1 study from multi-center. In these studies, antibiotic susceptibility of *M. leprae* included the mouse footpad assay, direct sequencing, and the quantitative PCR high‐resolution melting (qPCR‐HRM). All selected articles were observational in nature. Additionally, we examined codon mutations associated with the drug resistance-determining region (DRDRs) in genes like *folP1*, *rpoB*, *gyrA*, and *gyrB* which are known as drug resistance-inducing genes in *M. leprae*. Our statistical synthesis encompassed data from 4,454 leprosy patients, which categorized in four groups including: new cases, monotherapy patients, patients treated with MDT regimen, and relapse cases.

### 3.3 Prevalence of DR *M. Leprae*

**Fig. 2:**
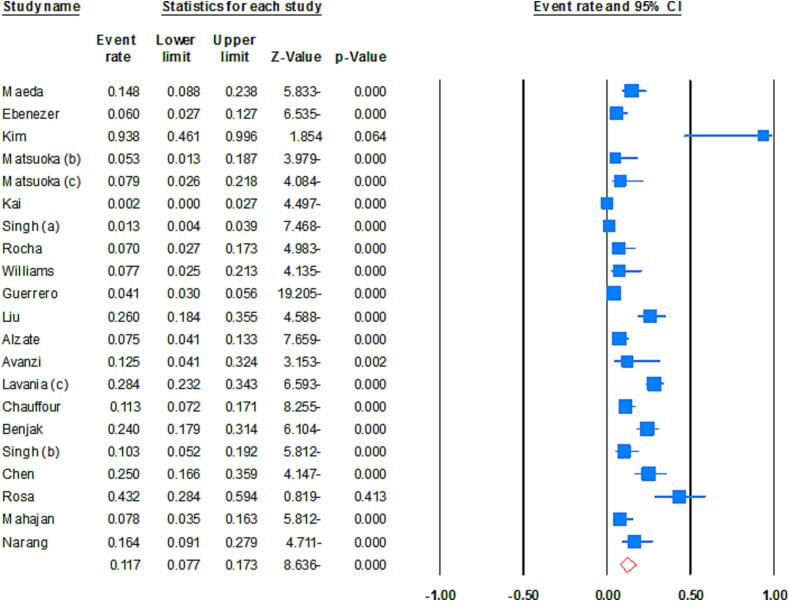
Forrest plots of the meta-analysis on the prevalence of DR *M. leprae*.

The combined prevalence of DR *M. leprae* was approximately 11.7 % (95 % CI: 7.7–17.3; *I^2^*: 90.79; *p* value: 0.01; Egger’s *p* value: 0.12; Begg's *p* value: 0.06) (Fig. 2). We identified two studies that could potentially contribute to heterogeneity. Nevertheless, after performing a sensitivity analysis, the pooled estimates showed minimal changes and maintained the reduced heterogeneity rate. According to the WHO regional classification, the prevalence of DR *M. leprae* was estimated as follows: 16.5 % in WPR, 12.6 % in SEAR, 7.5 % in AMR, 16.9 % in EUR, and 12.5 % in AFR. In terms of specific drug-resistance, the combined estimates demonstrated a 7.4 % resistance rate to dapsone, 5.1 % to rifampin, 1.8 % to clofazimine, and 3.5 % to ofloxacin. The prevalence rates of DR *M. leprae* were 20.9 % prior to 2009, 3.3 % during 2010–2012, 9.7 % during 2013–2016, and 19.3 % for the years 2017–2021. These results show stable trend in the prevalence of DR *M. leprae* throughout the current two decades.

### 3.4 The prevalence of MDR *M. Leprae*

**Fig. 3:**
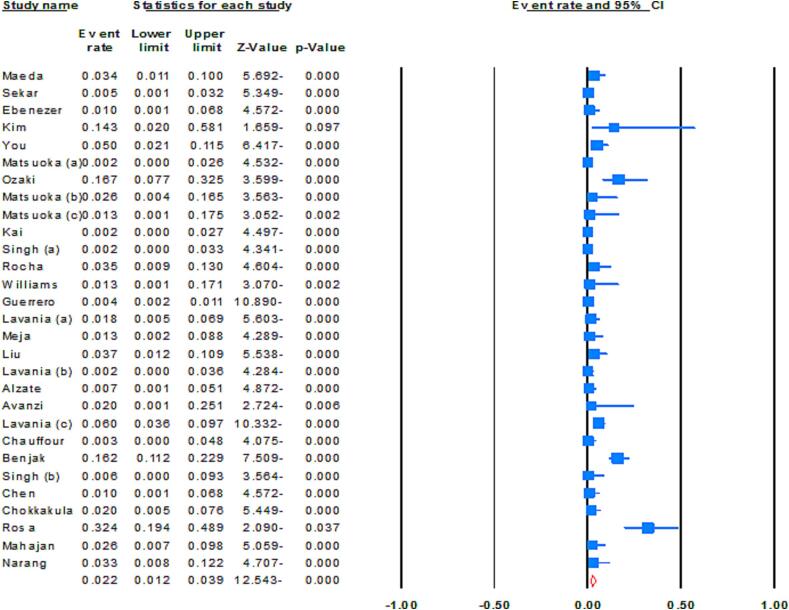
Forrest plots of the meta-analysis on the prevalence of MDR *M. leprae*.

Based on our synthesized findings the global prevalence rate of MDR *M. leprae* was 2.2 % (95 % CI: 1.2–3.9; *I^2^*: 82.68; *p* value: 0.01; Egger’s *p* value: 0.01; Begg’s *p* value: 0.03) (Fig. 3). In order to identify the underlying causes of heterogeneity, the impact of each individual study on the global estimates was carefully examined. This was achieved by reconducting the meta-analysis, each time excluding a different individual study. The sensitivity analysis confirmed the robustness of our initial findings.

**Fig. 4:**
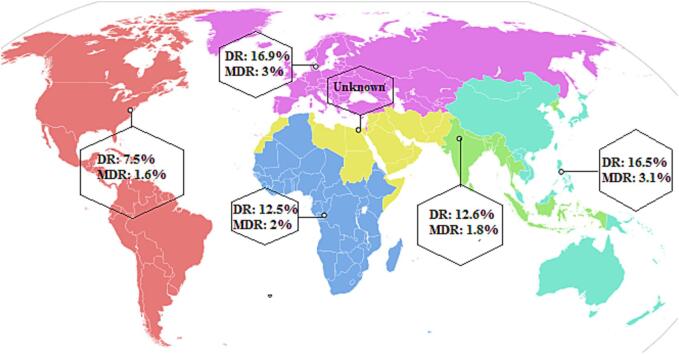
Geographical distribution of Drug-resistant *M. leprae* based on WHO region.

Upon further analysis, the prevalence of MDR *M. leprae* among all detected DR *M. leprae* cases was estimated at 23.2 % (95 % CI: 13.5–36.9; *I^2^*: 69.75; *p* value: 0.01; Egger’s *p* value: 0.09; Begg’s *p* value: 0.41). A significant proportion of these MDR *M. leprae* cases included leprosy patients with a history of previous treatments and those identified as relapse cases. Considering this, systematic evaluation of DR *M. leprae* is very important to detect and subsequently prevent proliferation of MDR *M. leprae* strains. In context with the WHO regional classifications, the highest rate of MDR *M. leprae* was observed form the WHO WPR and the WHO EUR, with 3.1 % and 3.0 %, respectively, across all global regions. The ensuing rates for other regions were: 2.0 % (95 %CI: 0.1–25.1) in WHO AFR, 1.8 % (95 % CI: 0.8–4.1) in the WHO SEAR and 1.6 % (95 % CI: 0.3–9.0) in the WHO AMR (Fig. 4).

**Fig. 5:**
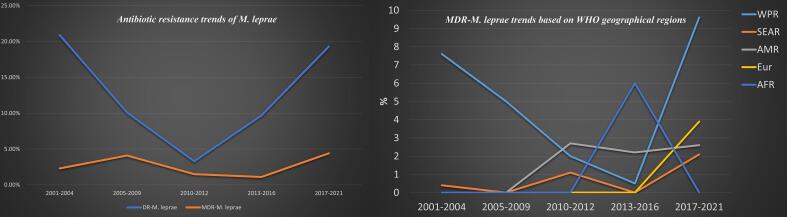
Epidemiological trends of DR *M. leprae*, MDR *M. leprae* as well as MDR-*M*. *leprae* related trend based on the geographical distribution over the recent two decades.

Subsequently, temporal trends of MDR *M. leprae* infections were assessed in five distinct time periods: 2001–2004, 2005–2009, 2010–2012, 2013–2016, and 2017–2021. The prevalence rate obtained was as follows: 2.3 % during 2001–2004, 4.1 % during 2005–2009, 1.5 % during 2010–2012 phase, 1.1 % during 2013–2016, and 4.4 % during 2017–2021. A comprehensive review of these findings clearly indicates an almost steady trend in the global prevalence of MDR *M. leprae* over the past two decades; however, there is notable increase in MDR *M*. *leprae* trend in geographical regions i.e., WHO Region of the Americas as well as European countries (Fig. 5).

### 3.5 Associative risk factors with DR *M. Leprae*

**Fig. 6:**
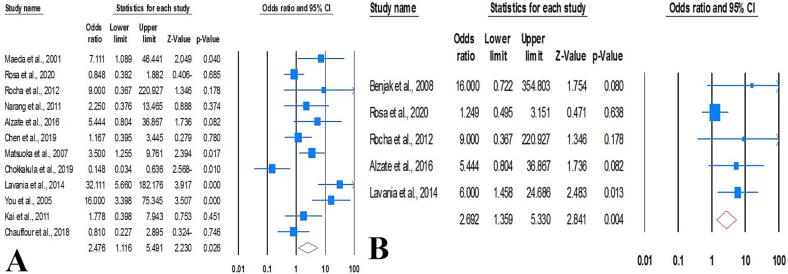
Forrest plots of the meta-analysis on the risk factor associated with DR *M. leprae*; A) lepromatous form in comparison with tuberculoid form, B) multibacillary form in comparison with paucibacillary form.

Bacterial density plays a decisive role in the risk of drug resistance among leprosy patients. Our subgroup analysis distinctly elucidated a significantly augmented risk of DR *M. leprae* in ML cases when juxtaposed with PB subtypes (OR: 2.69; 95 % CI: 1.35–2.48; *I^2^*: 35.65; *p* value: 0.1; Egger’s *p* value: 0.06; Begg’s *p* value: 0.5). It is also important to emphasize that the risk of DR *M. leprae* is significantly exacerbated in the lepromatous phase compared to patients showing characteristics of tuberculosis (OR: 1.7; 95 % CI: 0.5–5.78; *I^2^*: 0.0; *p* value: 0.4; Egger’s *p* value: 0.1; Begg’s *p* value: 0.6). Therefore, the density of *M. leprae* colonization and its lepromatous manifestations are significantly intertwined with the risk propensity of DR *M. leprae*. However, there is no significant association between treatment history and DR *M. leprae* infections (OR: 0.30; 95 % CI: 0.02–4.52; *p* value: 0.35; *I^2^*: 83.14; *p* value: 0.01; Egger’s *p* value: 0.48; Begg’s *p* value: 0.5). Our findings suggest that drug history also plays an important role as a risk factor for DR *M. leprae* infections. Nevertheless, recurrent cases were undeniably associated with an increased risk of DR *M. leprae* (Fig. 6).

To decipher the root of the heterogeneity, a meta-regression analysis was performed that assessed the potential influence of confounding variables. As delineated in Table 2, numerous moderating variables, including gender, geographical latitude, publication year, and participant sample size (either < 100 or > 100), substantially influence the heterogeneity of overall estimates.

**Table 2:** Meta-regression analysis of moderator variables influencing the overall estimates regarding *DR M. leprae*.

Moderators	Coefficient	SE	95 % CI	*P* value
Sample size	0.39	0.139	0.12–0.66	0.01
Year of publication	0.06	0.01	0.04–0.08	0.01
Method	1.72	0.07	1.86–1.58	0.5
Latitude	0.13	0.05	0.24–0.02	0.01
sex	0.47	0.13	0.73–0.21	0.01

### 3.6 Publication bias

**Fig. 7:**
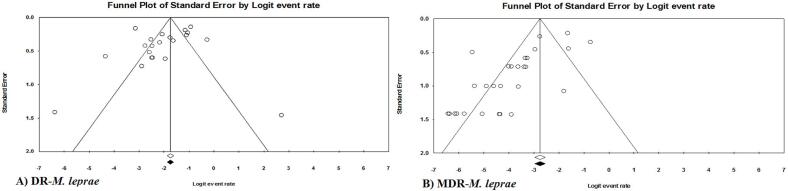
Funnel plots depicting publication bias of studies reporting the antimicrobial resistance prevalence in *M. leprae*; A) regarding the DR-*Mycobacterium leprae*; B) MDR-*Mycobacterium leprae*.

This research effort also required careful consideration of potential publication bias, using both Begg’s and Egger’s *p* value methods. Specific cases that showed significant bias were confirmed through funnel plot analysis. The asymmetry of the funnel plot lends support to the theory that studies with positive results may have a publication bias. Nevertheless, in instances of pronounced publication bias, the trim-fill technique was implemented. After this correction, the resulting consolidated estimates did not have any significant deviations from the initial estimates. Thus, the matching posts on the application of the fill-in method emphasize that any publication bias had an insignificant effect on the overall estimates (Fig. 7).

## 4 Discussion

The results of the present meta-analysis provide a comprehensive understanding of the risk factors associated with drug-resistant *M. leprae* and show the importance of bacterial density and disease subtype in determining susceptibility. One of the salient findings of our research is the increased risk of DR *M. leprae* among ML cases compared to PB subtypes. A plausible explanation for these observations is based on the underlying pathology of the disease. The bacterial load in MB cases is typically more than five skin lesions, nerve involvement, or the presence of bacilli in a slit-skin smear, while PB cases have one to five skin lesions without bacilli evident on skin smears [5]. Thus, ML cases inherently have a higher bacterial load, which increases the likelihood of drug-resistant strains [35]. Furthermore, the dynamics of drug resistance in leprosy are compounded by treatment regimens and duration. ML leprosy typically requires a more prolonged and intensive treatment course compared to PB leprosy. This extended exposure to antibiotics can further select for resistant strains in a high-bacterial-load environment [42]. This insight contributes to the broader research on *M. leprae*, highlighting the need for more nuanced approaches to managing and researching leprosy, especially in contexts of varying bacterial burdens. In addition, the apparent increased risk of DR *M. leprae* during the lepromatous phase, compared to the tuberculoid form, could be explained by an increase in the previously normal bacterial density [43].

Our study shows a clear association between the risk of DR *M. leprae* and certain aspects of medical history, especially in relapse cases; recurrent cases may have been exposed to multiple rounds of medication, which increases the likelihood of the emergence and proliferation of resistant strains. It highlights the importance of personalized treatment approaches, considering the patient’s medical history, and underlines the necessity of ongoing surveillance and research to monitor the efficacy and safety of current treatment regimens in the face of evolving bacterial resistance. Each additional round of medication, especially if not completed or if the regimen is suboptimal, potentially amplifies the risk of developing and propagating resistant strains of *M. leprae* [44]. This phenomenon is rooted in the fundamental principles of antibiotic resistance, where repeated and prolonged exposure to antimicrobial agents can select for bacterial strains that have developed mechanisms to survive these treatments [45], [46]. In a meta-analysis conducted by Lazo-Porras et al. they found that there was no significant difference between WHO MDT and rifampin-ofloxacin-minocycline regimens from aspect like complete cure, clinical improvement, treatment failure, relapse rate, or type I reaction in PB or MB leprosy patients [47]. However, their study also brings to light an essential consideration: the need for more comprehensive research to evaluate the long-term outcomes and potential adverse effects of the WHO regimen. While their results show that the WHO MDT works in the short term, the fact that bacterial resistance is always changing, as shown in our study, means that treatment protocols need to be constantly reviewed and changed.

Nevertheless, it was interesting to note that new untreated cases and previous mono-therapies did not show a significant association with DR *M. leprae*. This finding is intriguing as it suggests that resistance is less likely to develop in patients who have not been previously treated or were treated with a single drug, emphasizing the critical role of combination drug therapy in effectively managing leprosy and containing the emergence of resistance. *M. leprae* is a slow-growing pathogen that is hard to treat. To stop resistance from forming, which usually happens because of bad treatment methods like not giving the right amount of medicine, not finishing the treatment course, or only using one therapy, you need to use a variety of methods. In addition, a meta-regression analysis explored the effect of several moderating variables like gender, geographical latitude, publication year, and sample size that could influence our findings [48]. Their study highlights the crucial role of efflux pumps, which are proteins that bacteria use to expel toxic substances, including antibiotics, from their cells [49]. These efflux pumps are not only instrumental in developing drug resistance but also play a key role in the intracellular survival of *M. leprae* and its adaptation to the host environment. Understanding these mechanisms is essential for developing new therapeutic strategies and anticipating the evolution of resistance patterns. An interesting distinction between *M. tuberculosis* and *M. leprae* lies in their efflux pumps; for example, *M. leprae* lacks the MmpS5-MmpL5 efflux pump, which could explain its vulnerability to clofazimine [50].

Our meta-analysis highlighted a significant incidence of antibiotic resistance in *M. leprae* strains. Specifically, the pooled incidence of *M. leprae* resistance to rifampicin was estimated to be 11 % (95 % CI, 7 % to 15 %). This indicates that more than one in ten cases could involve a strain of *M. leprae* that is resistant to this crucial antibiotic. Moreover, our results suggest notable regional variations in resistance patterns. The WPR emerged as a region of particular concern, showcasing the highest rate of rifampicin resistance at 21 %. This finding is concerning, as it suggests that current treatment regimens might be less effective in this region. In contrast, the AMR presented a relatively lower incidence, recorded at 4 %. Such geographic disparities in resistance profiles emphasize the need for regionally specific strategies and interventions [6]. Another meta-analysis study found that the global drug-resistance rate to MDT therapies was 10.18 %, and the highest rate was in the WPR with 17.05 % [3]. This convergence of evidence from multiple studies highlights the urgent need for enhanced surveillance, the development of new treatment options, and the implementation of targeted interventions in regions with high resistance rates.

The escalating prevalence of MDR *M. leprae* worldwide is a growing concern, underscoring the urgency to reassess and potentially revise existing treatment protocols. This resistance may stem from a variety of factors, including inconsistent or inadequate treatment courses, reduced efficacy of drugs, and the pathogen's inherent adaptation abilities [50], [51]. It's crucial to acknowledge the significant impact of advancements in molecular detection technology and the training of professionals in this domain. These advancements have markedly improved the detection and understanding of resistance rates. Molecular diagnostic methods are notable for their speed and sensitivity in identifying resistance genes, playing a pivotal role in the investigation of antimicrobial resistance [52]. These assays, offering more accurate and rapid results, stand in contrast to traditional phenotypic methods that depend on monitoring microorganism growth in antibiotic environments. As our comprehension of antimicrobial resistance's molecular mechanisms deepens, we can expect the development of even more sophisticated molecular detection techniques [53]. It is essential to continuously update and refine these methods to ensure they provide relevant, precise resistance detection in clinical microbiology settings. The heightened resistance observed in specific regions and time periods further highlights the importance of regional healthcare policies, treatment adherence, and possible genetic variations in the pathogen. Advantages of the study include a rigorous meta-regression analysis that sheds light on potential confounders such as gender, latitude, publication year, and sample size.

This comprehensive approach provides a detailed understanding of the various factors influencing the results and allows for a stronger interpretation of the findings. However, there are limitations in our investigation, such as 1) low population size, 2) heterogeneity that was not significantly reduced after sensitivity analysis, and 3) the presence of publication bias among eligible studies. A prominent issue of persistent heterogeneity was observed, which could not be significantly reduced through sensitivity analysis. This lingering heterogeneity can be attributed to clinical, methodological, and statistical origins. Clinically, attributes such as the participant’s characteristics, the severity of *M. leprae* infection, socioeconomic status, body mass index (BMI), and gestational age could contribute to this variability. Statistical differences may emerge while performing subgroup analyses, leading to conflicting results. Such counterintuitive findings emphasize the multifaceted relationship between pathogens and host response that warrants further exploration. Future research efforts should delve deeper into understanding the genetic makeup of resistant strains and evaluate newer therapeutic combinations.

## 5 Conclusions

Our investigation provides a deepened insight into the escalating challenge posed by MDR *M. leprae*. The data highlight the increasing prevalence and risks associated with MDR *M. leprae*, which require an urgent global response. The regional variations in resistance identified in our study emphasize the need for tailored strategies to effectively address the nuances of this growing issue. The adaptability of *M. leprae*, especially its MDR strains, calls for an enhancement in our diagnostic precision and treatment approaches. By delving into the intricacies of MDR *M. leprae*, this study aims to serve as a foundation for refining therapeutic strategies, guiding targeted research, and informing public health policies to ensure optimized care for leprosy patients.

**Declarations**

**Ethics approval and consent to participate**

Not applicable (this paper was provided based on researching in global databases).

**Consent for publish**

Not Applicable.

**Availability of data and materials**

All data generated or analyzed during this study are included in this published article.

**Competing interests**

There is no any conflict of interest among the all authors.

**Funding**

We have not received any funding for this research.

**Authors' Contributions**

HZ has contributed to design of the work and analysis of data.

FA and MK have drafted the work and substantively revised it.

HZ, FA, and MK have reviewed and revised the draft manuscript:

All authors read and approved the final manuscript.

## CRediT authorship contribution statement

**Hamidreza Zivarifar:** Writing – original draft. **Forough Ahrari:** Writing – original draft. **Mohsen Karbalaei:** Writing – review & editing.

## Declaration of competing interest

The authors declare that they have no known competing financial interests or personal relationships that could have appeared to influence the work reported in this paper.
